# Validation of Sarcopenic Obesity Screening Tools: A Cross-Sectional Analysis Based on ESPEN and EASO Criteria

**DOI:** 10.3390/medicina61111909

**Published:** 2025-10-24

**Authors:** Seongmin Choi, Miji Kim, Yunsoo Soh, Chang Won Won

**Affiliations:** 1 Department of Rehabilitation Medicine, Seoul National University Bundang Hospital, Seoul National University College of Medicine, Seongnam 13620, Republic of Korea; 2 Department of Biomedical Science and Technology, College of Medicine, East-West Medical Research Institute, Kyung Hee University, Seoul 02453, Republic of Korea; 3 Department of Physical Medicine and Rehabilitation Medicine, Kyung Hee University Medical Center, Seoul 02447, Republic of Korea; 4 Department of Family Medicine, College of Medicine, Kyung Hee University, Kyung Hee University Medical Center, Seoul 02447, Republic of Korea

**Keywords:** screening validation, Korean frailty and aging cohort study, older adults

## Abstract

*Background and Objectives*: Sarcopenic obesity, characterized by sarcopenia and obesity, is associated with adverse outcomes. The recent consensus from the European Society for Clinical Nutrition and Metabolism (ESPEN) and the European Association for the Study of Obesity (EASO) proposed a diagnostic algorithm (screening, diagnosis, and staging) for sarcopenic obesity. However, the effectiveness of recommended screening tools for sarcopenic obesity remains unclear. This study aimed to assess the performance of SARC-F questionnaire (Strength, walking Assistance, Rise, Climb, and Falls), calf circumference (CC), and SARC-CalF (SARC-F combined with CC), combined with obesity indicators, as screening tools for sarcopenic obesity. *Materials and Methods*: This cross-sectional study analyzed 2020 community-dwelling older adults from the Korean Frailty and Aging Cohort Study. Sarcopenic obesity was defined according to ESPEN and EASO criteria. Screening tools included SARC-F, CC, and SARC-CalF in combination with high body mass index (BMI; ≥25 kg/m^2^) or high waist circumference (WC; men, ≥90 cm; women, ≥85 cm). The diagnostic performance was evaluated using sensitivity, specificity, and predictive value. *Results*: SARC-F (≥4) with high BMI or WC demonstrated low sensitivity (men, 5.68%; women, 17.82%) but high specificity (men, 99.03%; women, 94.35%) and negative predictive value (NPV) (men, 91.68%; women, 91.09%). Lowering the SARC-F cutoff improved sensitivity but reduced specificity. CC combined with a high BMI or WC showed modest sensitivity (men, 34.09%; women, 34.65%) but moderate specificity (men, 59.48%; women, 59.91%). SARC-CalF (≥11) combined with high BMI or WC showed slightly higher sensitivity (men, 13.64%; women, 19.80%) but lower specificity (men, 95.04%; women, 86.93%) than SARC-F. *Conclusions*: SARC-F combined with obesity indicators may serve as a case-finding tool with high specificity and NPV, supporting its usefulness in ruling out sarcopenic obesity in the clinical setting. Meanwhile, CC was not an effective screening tool, and SARC-CalF did not substantially improve sensitivity or accuracy compared with SARC-F.

## 1. Introduction

Loss of skeletal muscle mass and function generally occurs with aging, and it is often accompanied by an increase in body fat [1]. These age-related changes are driven by multiple mechanisms, including decreased levels of anabolic hormones, reduced physical activity, and chronic low-grade inflammation, which, together, promote muscle loss and fat accumulation. Anabolic resistance, mitochondrial dysfunction, and insulin resistance impair muscle protein synthesis, while elevated pro-inflammatory cytokines such as interleukin-6 and tumor necrosis factor-α exacerbate muscle catabolism and adipose tissue expansion [2]. This process promotes the development of sarcopenic obesity, a clinical condition characterized by the coexistence of sarcopenia and obesity [3]. Sarcopenic obesity is a strong risk factor for adverse clinical outcomes such as functional impairment, metabolic disease, and increased mortality [4,5]. However, information about sarcopenic obesity remains limited. One major reason for this limited information is the lack of standardized diagnostic criteria for sarcopenic obesity [6]. To address the heterogeneity in definitions and diagnostic approaches for sarcopenic obesity, the European Society for Clinical Nutrition and Metabolism (ESPEN) and the European Association for the Study of Obesity (EASO) recently developed the first international consensus on the diagnostic criteria for sarcopenic obesity [7]. According to the ESPEN and EASO consensus, diagnostic assessment is divided into three steps: initial screening, followed by diagnostic procedures for subjects with positive screening results, and then staging of the subjects diagnosed with sarcopenic obesity. Screening is based on a high body mass index (BMI) or waist circumference (WC), which are indicators of obesity and surrogate indicators of sarcopenia (e.g., clinical symptoms, suspicion factors, or validated questionnaires such as SARC-F), and body composition and skeletal muscle function are assessed for diagnosis [7].

Therefore, screening tools that can efficiently identify individuals requiring further diagnostic measurements should be validated. However, to the best of our knowledge, only one study has validated the screening tools proposed by ESPEN and EASO. A previous study evaluated a screening tool defined as the concomitant presence of a high BMI or WC and clinical suspicion factors for sarcopenia (e.g., history of disability, falls, metabolic syndrome, diabetes mellitus). Their results demonstrated high sensitivity (100%), low specificity (21.7%), and low overall accuracy (28.8%), indicating that further studies are needed to improve the specificity of screening tools to reduce unnecessary diagnostic procedures and workload of healthcare professionals [6].

Among the screening tools proposed by the ESPEN and EASO consensus, SARC-F has been widely investigated. This self-reported questionnaire consists of five items assessing strength, assistance in walking, rising from a chair, climbing stairs, and falls. Each item is scored from 0 to 2 points, with a maximum score of 10 points [8]. Previous studies have consistently shown a low sensitivity (14–21%) and high specificity (90–94%) for sarcopenia [9,10,11,12]. Because of its high specificity, many guidelines currently recommended it as a formal case-finding tool for sarcopenia [12,13,14,15]. However, as case finding and screening serve different purposes, where screening involves simple tools for the general population, while case finding focuses on high-risk individuals, it is important to evaluate the effectiveness of SARC-F specifically as a screening tool for sarcopenic obesity, which remains unstudied.

According to the Asian Working Group for Sarcopenia (AWGS) 2019 consensus, calf circumference (CC) and SARC-CalF are also recommended as case-finding tools for sarcopenia [13]. CC demonstrates moderate to high sensitivity and specificity in predicting sarcopenia [16,17,18], and SARC-CalF further improves the sensitivity of SARC-F by incorporating CC [19], thereby offering an advantage in identifying at-risk individuals. Based on this evidence, we hypothesized that CC and SARC-CalF could also be useful in screening for sarcopenic obesity.

Therefore, this study aimed to evaluate the performance of combining the SARC-F questionnaire, CC, and SARC-CalF with obesity indicators to screen for sarcopenic obesity.

## 2. Materials and Methods

### 2.1. Study Design and Population

**Figure 1:**
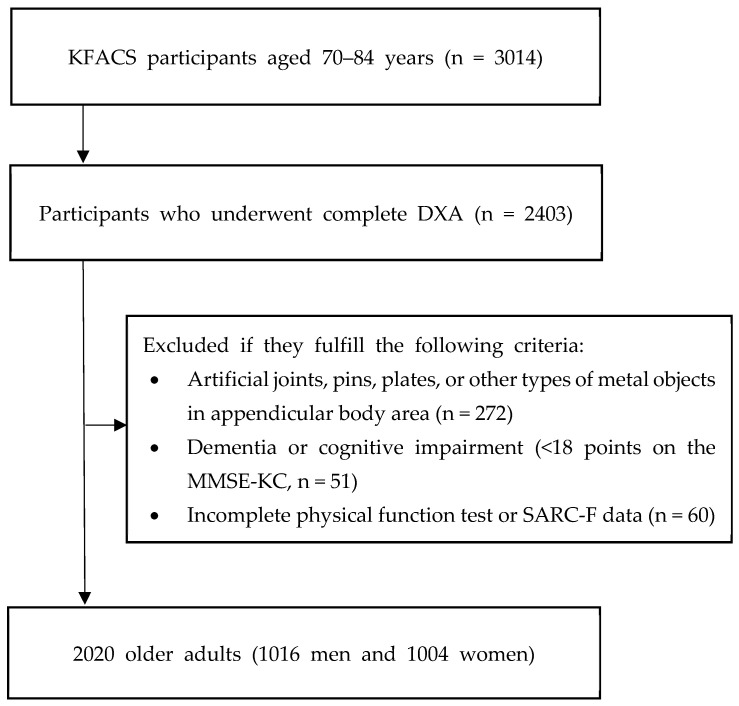
Flowchart of the participant recruitment process. KFACS, Korean Frailty and Aging Cohort Study; DXA, dual-energy X-ray absorptiometry; MMSE-KC, Mini-Mental State Examination in the Korean version of the Consortium to Establish a Registry for Alzheimer’s Disease assessment packet.

The study participants consisted of adults aged 70–84 years who participated in the baseline survey of the Korean Frailty and Aging Cohort Study (KFACS) between 2016 and 2017. The KFACS is a nationwide cohort study designed to identify risk factors for frailty and aging in community-dwelling older adults. This study employed a cross-sectional design based on the baseline data of the KFACS. Of the 3014 participants enrolled during the 2016–2017 period, 2403 who underwent complete dual-energy X-ray absorptiometry (DXA) were included. The final analysis included 2020 participants (men, 1016; women, 1004) after excluding 272 participants with artificial joints, pins, plates, or other types of metal objects in appendicular body regions, 51 participants with dementia or moderate to severe cognitive impairment (<18 points on the Mini-Mental State Examination in the Korean version of the Consortium to Establish a Registry for Alzheimer’s Disease assessment packet), and 60 participants with incomplete physical function test or SARC-F questionnaire data (Figure 1).

In this study, the primary variables of interest were the screening tools for sarcopenic obesity (SARC-F, calf circumference, and SARC-CalF). The reference standard was sarcopenic obesity defined according to the ESPEN and EASO consensus criteria. Additional variables such as age, sex, and comorbidities were collected to describe participant characteristics.

To minimize potential bias, all study sites followed a standardized operating procedure. Trained investigators performed anthropometric and functional assessments using identical measurement protocols.

The KFACS protocol was approved by the Institutional Review Board (IRB) of the Clinical Research Ethics Committee of Kyung Hee University Medical Center (IRB number: 2015-12-103). All participants signed an informed consent form before participation.

### 2.2. Sarcopenic Obesity

Sarcopenic obesity was evaluated according to the ESPEN and EASO diagnostic criteria [7]. Participants with low appendicular lean mass (ALM), high fat mass, and either an abnormal Five Times Sit-to-Stand Test score or low handgrip strength (HGS) were defined as having sarcopenic obesity. Because the ESPEN and EASO consensus recommends ethnicity-specific cutoff values for each parameter, we applied those corresponding to Asian values. Body composition was assessed using DXA. ALM was adjusted for body weight (ALM/W), and the cutoff values were <29.5% for men and 23.2% for women [20]. Body fat mass was also adjusted to body weight (body fat mass/weight), and the cutoff values were >29.7% for men and >37.2% for women [21]. HGS was measured using a hand dynamometer (T.K.K. 5401; Takei Scientific Instruments Co., Ltd., Tokyo, Japan). We instructed the participants to squeeze the handle maximally, with the elbows extended, in standing position, twice on both sides, and obtained the maximum value in kilograms. The cutoff values were <28 kg for men and 18 kg for women [13]. The Five Times Sit-to-Stand Test was performed by asking the participants to rise from a chair and sit down five times as quickly as possible, and the time was recorded. The cutoff value was ≥17 s [22]. Trained investigators performed all assessments.

### 2.3. Screening Tools for Sarcopenic Obesity

Positive screening was defined according to the ESPEN and EASO consensus criteria. Participants with the concomitant presence of either high BMI or WC and suspected factors for sarcopenia (e.g., SARC-F, CC, SARC-CalF) were defined as having a positive screening result. Ethnicity-specific cutoff values were applied to each parameter.

#### 2.3.1. Body Mass Index and Waist Circumference

BMI was calculated as body weight (kg) divided by height squared (m^2^). The cutoff value was ≥25 kg/m^2^ according to the Asia-Pacific criteria of the World Health Organization guidelines [23]. WC was measured at the midpoint between the lower margin of the 12th rib and the iliac crest, with cutoff values of ≥90 cm for men and ≥85 cm for women, as defined by the Korean Society for the Study of Obesity [24].

#### 2.3.2. Calf Circumference

To measure CC, the participants were instructed to stand upright with their feet shoulder-width apart. In this position, the circumference of the widest part of the calf was measured using an inelastic tape. The cutoff values were <34 cm for men and <33 cm for women [13].

#### 2.3.3. SARC-F (Strength, Walking Assistance, Rise, Climb, and Falls)

The SARC-F questionnaire consists of five items (strength, assistance in walking, rising from a chair, climbing stairs, and falls), each scored from 0 to 2 points, with a maximum total of 10 points. A score ≥4 was considered the cutoff value [8]. The questionnaire was administered through face-to-face interviews by trained staff.

#### 2.3.4. SARC-CalF

SARC-CalF combines CC with SARC-F and assigns equal weights to SARC-F (10 points) and low CC (10 points). A score ≥11 was used as the cutoff value for screening [13,25].

### 2.4. Other Measurements

Trained investigators obtained sociodemographic data and medical histories, including age, sex, height, weight, comorbidities, and smoking and alcohol consumption status. Smokers were defined as participants who smoked at least one cigarette per week, whereas alcohol consumers were defined as those who consumed alcohol more than once per week.

### 2.5. Statistical Analyses

Continuous variables were compared using the independent t-test or Mann–Whitney U test, and Pearson’s chi-square test was used to compare categorical variables. Categorical variables are presented as numbers and ratios (%), and continuous variables are expressed as means ± standard deviations. The performance of each screening tool, including sensitivity, specificity, positive predictive value (PPV), negative predictive value (NPV), and accuracy, was calculated using the Diagnostic Test Evaluation Calculator (version 20.210) provided by MedCalc Software Ltd. (https://www.medcalc.org/calc/diagnostic_test.php, accessed on 4 November 2024) [6] and expressed as percentages. The predictive power of each screening tool was evaluated using receiver operating characteristics (ROC) curve analysis. Statistical analyses were performed using the Statistical Package for Social Sciences version 25.0 (IBM Corp., Armonk, NY, USA), and *p* < 0.05 was considered statistically significant.

## 3. Results

The baseline characteristics of the participants according to sex are presented in Table 1. Among the 2020 participants, 1016 (50.3%) were men, and 1004 (49.7%) were women. Men had significantly higher body weight, HGS, ALM/W, WC, and CC but lower body fat mass than women. The mean SARC-F (men, 0.60 ± 1.05; women, 1.40 ± 1.58) and SARC-CalF scores (men, 4.60 ± 5.04; women 5.36 ± 5.33) were significantly higher in women than in men. The proportion of participants with positive screening results on all tools combining SARC-F, CC, or SARC-CalF with high BMI or WC was significantly higher in women than in men. The prevalence of sarcopenic obesity did not differ significantly between sexes (men, 8.7%; women, 10.1%).

Table 2 presents the performance of each screening assessment. Positive screening defined as concomitant presence of SARC-F (≥4) and either high BMI or WC demonstrated low sensitivity (men, 5.68%; women, 17.82%) but high specificity (men, 99.03%, women, 94.35%) and high NPV (men, 91.68%; women, 91.09%) in both sexes. Screening for CC combined with high BMI or WC showed modest sensitivity (men, 34.09%; women, 34.65%), moderate specificity (men, 59.48%; women, 59.91%), and moderate accuracy (men, 57.27%; women, 57.36%). In addition, SARC-CalF (≥11) combined with high BMI or WC showed slightly higher sensitivity (men, 13.64%; women, 19.80%) but lower specificity (men, 95.04%; women 86.93%) than SARC-F–based screening in both sexes.

**Figure 2:**
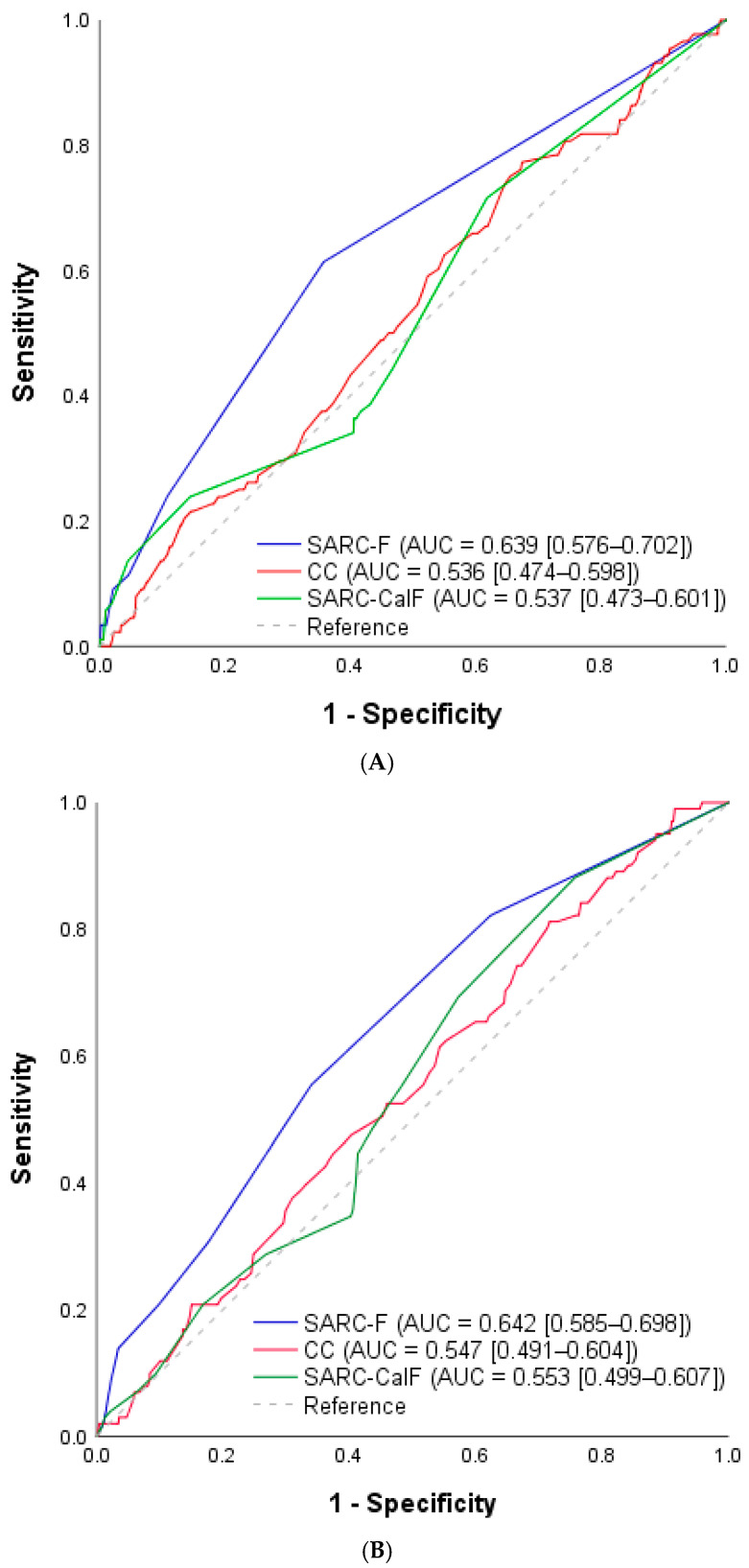
Receiver operating characteristic curve analysis of SARC-F, SARC-CalF, and CC: (**A**) men and (**B**) women. AUC, area under the curve; CC, calf circumference.

ROC curve analysis was performed to evaluate the predictive power of SARC-F, SARC-CalF, and CC as screening tools for sarcopenic obesity (Figure 2). Among the tools tested, SARC-F demonstrated the most effective performance, showing modest but statistically significant predictive power in both men (area under the curve [AUC] = 0.639, 95% confidence interval [CI]: 0.576–0.702) and women (AUC = 0.642, 95% CI: 0.585–0.698). In contrast, CC and SARC-CalF in both men and women did not yield significant results.

Because of the low sensitivity observed with SARC-F (≥4) combined with high BMI or WC, we additionally analyzed the performance of different SARC-F cutoff values (Table 3). Lowering the cutoff increased the sensitivity but decreased the specificity in both sexes.

## 4. Discussion

In this study, we investigated the performance of SARC-F, CC, and SARC-CalF combined with high BMI or WC as screening tools for sarcopenic obesity according to the ESPEN and EASO consensus. Our results showed that SARC-F combined with high BMI or WC had low sensitivity but high specificity and high NPV in identifying sarcopenic obesity. In contrast, CC was not effective in predicting sarcopenic obesity, and SARC-CalF did not substantially improve the sensitivity or overall diagnostic accuracy compared with SARC-F.

Our findings are consistent with previous reports showing that SARC-F generally has low sensitivity but high specificity for sarcopenia [12,26,27]. Although SARC-F demonstrates low sensitivity, its high specificity has led many sarcopenia guidelines to recommend it as a case-finding tool that requires further evaluation [13,14,15]. Although the ESPEN and EASO consensus emphasizes that screening tools should prioritize sensitivity [7], our results highlight that SARC-F combined with high BMI or WC offers high specificity, suggesting it may be better suited as a case-finding tool to help reduce unnecessary device-based measurements and healthcare workload.

In addition, the SARC-F is simple, inexpensive, and can be completed within one minute without specialized equipment, making it highly feasible for use in primary care and community settings [8]. Its practicality allows early identification of older adults at risk of sarcopenic obesity, facilitating referral for confirmatory assessment.

To improve the sensitivity of SARC-F, we analyzed alternative cutoff values and found that lowering the threshold increased sensitivity but decreased specificity. Consistent with our findings, several previous studies have reported similar trends when evaluating different SARC-F thresholds for sarcopenia [28,29,30]. The AWGS 2019 guideline recommends a SARC-F cutoff value of ≥4, whereas other sarcopenia guidelines mention SARC-F as a screening tool without specifying cutoff values [13,14,15]. Additionally, a recent meta-analysis suggested that using a lower cutoff value (≥1) may improve sensitivity, as undiagnosed sarcopenia can have more serious consequences [9]. Thus, when using SARC-F combined with high BMI or WC, exploring different cutoff thresholds may help balance sensitivity and specificity.

Previous Asian validation studies have reported that CC performs well in diagnosing sarcopenia (sensitivity, 76–88%; specificity, 64–91%) [16,17,31], and the ASWG 2019 guidelines therefore recommend CC as a case-finding tool for sarcopenia. In contrast, our study showed that CC combined with high BMI or WC was not effective for screening sarcopenic obesity due to low sensitivity and specificity. This difference compared to CC’s performance in sarcopenia may be explained by the buildup of fat within the calf muscles of obese individuals [32]. Similarly, Lim et al. showed that the diagnostic accuracy of CC is reduced in sarcopenic obesity compared to non-obese sarcopenia (non-obese sarcopenia: AUC = 0.799, 95% CI: 0.716–0.881; sarcopenic obesity: AUC = 0.657, 95% CI: 0.556–0.758) [33].

The AWGS 2019 guidelines also recommend SARC-CalF as a case-finding tool for sarcopenia. Previous studies have reported that the SARC-CalF improves sensitivity of SARC-F by incorporating CC (sensitivity, 45.9–57.2%; specificity, 87.7–91.3%) [9,34,35]. However, in our study, SARC-CalF combined with high BMI or CC did not substantially improve the sensitivity of SARC-F, likely because of the limited predictive performance of CC for sarcopenic obesity.

This study has some limitations. First, we conducted a cross-sectional analysis limited to evaluating the performance of SARC-F, CC, and SARC-CalF, without validating the clinical suspicion factors for sarcopenia recommended by the EASO and ESPEN consensus. Second, the participants were ambulatory, community-dwelling older adults; therefore, the results may not be applicable to other populations. Third, as this study was conducted in an Asian population, the findings and the optimal SARC-F cut-off value may be difficult to generalize to non-Asian populations. Finally, because of the cross-sectional study design, we could not assess longitudinal outcomes such as functional decline, hospitalization, or mortality in relation to the screening tools.

Despite these limitations, this study has several strengths. First, it was performed in a large nationwide cohort of 2020 older adults. Second, this was the first study to evaluate the validity of SARC-F combined with high BMI or WC, as suggested by the ESPEN and EASO consensus.

Future studies should directly compare the performance of SARC-F with the clinical suspicion factors for sarcopenic obesity (e.g., history of disability, falls, metabolic syndrome, diabetes mellitus) proposed by the EASO and ESPEN consensus to determine which approach provides greater screening accuracy. Moreover, further research is needed to investigate whether integrating such clinical suspicion factors with the SARC-F can further improve the sensitivity, specificity, and overall diagnostic utility of screening for sarcopenic obesity. In addition, future research should assess the practicality and usefulness of such screening approaches in real-world clinical settings, including implementation studies to evaluate their feasibility, cost-effectiveness, and impact on clinical decision-making and patient outcomes.

## 5. Conclusions

SARC-F (≥4) combined with high BMI or WC demonstrated high specificity and high negative predictive value, suggesting its potential usefulness as a case-finding tool for efficiently ruling out sarcopenic obesity in clinical practice. Given its simplicity, low cost, and brief administration time, the SARC-F may be feasible for implementation in primary care and community-based settings. In contrast, CC combined with high BMI or WC may not be an effective screening tool, and SARC-CalF did not substantially improve sensitivity or accuracy compared with SARC-F. When applying SARC-F as a screening tool, alternative cutoff values below the conventional threshold of 4 may also be considered to improve sensitivity. Future studies are needed to assess its practicality and usefulness in real-world clinical settings.

## Figures and Tables

**Table 1:** Baseline characteristics of the participants according to sex.

	Men (n = 1016)	Women (n = 1004)	Total (n = 2020)	*p* Value
Age (years)	76.4 ± 3.9	75.4 ± 3.9	75.9 ± 3.9	<0.01 *
Height (cm)	165.0 ± 5.6	152.0 ± 5.2	158.5 ± 8.4	<0.01 *
Weight (kg)	65.3 ± 8.9	56.7 ± 7.6	61.09± 9.4	<0.01 *
Hypertension	540 (53.1)	582 (58.0)	1122 (55.5)	0.29
Dyslipidemia	248 (24.4)	418 (41.6)	666 (33.0)	<0.01 *
Diabetes mellitus	251 (24.7)	193 (19.2)	444 (22.0)	<0.01 *
Knee osteoarthritis	109 (10.7)	291 (29.0)	400 (19.8)	<0.01 *
Depression	19 (1.9)	32 (3.2)	51 (2.5)	0.06
Cardiovascular diseases	58 (5.7)	31 (3.1)	89 (4.4)	<0.01 *
Alcohol consumption	523 (51.5)	110 (11.0)	633 (31.3)	<0.01 *
Current smoking	110 (10.8)	10 (1.0)	120 (5.9)	<0.01 *
Diagnostic assessments of SO				
HGS (kg)	32.47 ± 5.71	21.27 ± 3.85	26.90 ± 7.43	<0.01 *
Five Times Sit-to-Stand Test (s)	10.47 ± 2.99	11.81 ± 4.05	11.14 ± 3.62	<0.01 *
ALM/W	29.56 ± 3.21	23.91 ± 2.89	26.75 ± 4.16	<0.01 *
Fat mass (%)	0.27 ± 0.06	0.37 ± 0.06	0.32 ± 0.08	<0.01 *
SO (%)	88 (8.7)	101 (10.1)	189 (9.4)	0.28
Screening assessments of SO				
BMI (kg/cm^2^)	23.96 ± 2.73	24.48 ± 2.82	24.22 ± 2.85	<0.01 *
WC (cm)	88.63 ± 8.45	86.13 ± 8.14	87.39 ± 8.39	<0.01 *
CC (cm)	34.34 ± 2.82	33.38 ± 2.88	33.86 ± 2.89	<0.01 *
SARC-F score	0.60 ± 1.05	1.40 ± 1.58	1.00 ± 1.40	<0.01 *
SARC-CalF score	4.60 ± 5.04	5.36 ± 5.33	4.97 ± 5.20	<0.01 *
SARC-F (≥4) + high BMI or WC ^1^	14 (1.4)	69 (6.9)	83 (4.1)	<0.01 *
CC (<Ref. ^2^) + high BMI or WC	149 (14.7)	194 (19.3)	343 (17.0)	<0.01 *
SARC-CalF (≥11) + high BMI or WC	58 (5.7)	138 (13.7)	196 (9.7)	<0.01 *

SO, sarcopenic obesity; HGS, hand grip strength; ALM/W, appendicular lean mass/weight; BMI, body mass index; WC, waist circumference; CC, calf circumference. ^1^ High BMI: ≥25 kg/m^2^; high WC: ≥90 cm for men and ≥85 cm for women. ^2^ Reference: <34 cm for men and <33 cm for women. * *p* < 0.05

**Table 2:** Screening assessments validated against sarcopenic obesity.

	Sensitivity (%)	Specificity (%)	PPV (%)	NPV (%)	Accuracy (%)
SARC-F (≥4) + high BMI or WC ^1^					
Men	5.68	99.03	35.83	91.68	90.91
Women	17.82	94.35	26.17	91.09	86.62
CC ^2^ + high BMI or WC					
Men	34.09	59.48	7.42	90.45	57.27
Women	34.65	59.91	8.85	89.08	57.36
SARC-CalF (≥11) + high BMI or WC					
Men	13.64	95.04	20.56	92.12	88.04
Women	19.80	86.93	14.55	90.61	80.15

PPV, positive predictive value; NPV, negative predictive value; BMI, body mass index; CC, calf circumference; WC, waist circumference. ^1^ High BMI: ≥25 kg/m^2^; high WC: ≥90 cm for men and ≥85 cm for women. ^2^ Reference: <34 cm for men and <33 cm for women.

**Table 3:** Validity of different SARC-F cutoffs with high BMI or WC for sarcopenic obesity.

	Sensitivity (%)	Specificity (%)	PPV (%)	NPV (%)	Accuracy (%)
Sarc-F (≥6) + high BMI or WC ^1^					
Men	2.27	99.89	66.77	91.47	91.40
Women	8.08	98.45	36.93	90.51	89.32
Sarc-F (≥5) + high BMI or WC					
Men	3.37	99.78	59.82	91.55	91.40
Women	15.49	97.79	44.01	91.15	89.47
Sarc-F (≥4) + high BMI or WC					
Men	5.68	99.03	35.83	91.68	90.91
Women	17.82	94.35	26.17	91.09	86.62
Sarc-F (≥3) + high BMI or WC					
Men	6.82	98.83	28.67	91.72	90.42
Women	24.75	90.03	21.81	91.42	83.44
Sarc-F (≥2) + high BMI or WC					
Men	15.91	95.26	24.23	92.24	88.36
Women	42.57	79.96	19.27	92.53	76.18
Sarc-F (≥1) + high BMI or WC					
Men	44.32	83.19	20.08	94.00	79.81
Women	59.41	61.02	14.62	93.05	60.86

PPV, positive predictive value; NPV, negative predictive value; BMI, body mass index; WC, waist circumference. ^1^ High BMI: ≥25 kg/m^2^; high WC: ≥90 cm for men and ≥85 cm for women.

## Data Availability

All cohort data supporting the findings of this study are available from the KFACS and are open to all researchers upon reasonable request. All news articles published in the KFACS database, data provision manuals, and contact information are available on the KFACS website (http://www.kfacs.kr).

## Abbreviations

The following abbreviations are used in this manuscript:

ESPEN	European Society for Clinical Nutrition and Metabolism
EASO	European Association for the Study of Obesity
CC	Calf circumference
BMI	Body mass index
WC	Waist circumference
AWGS	Asian Working Group for Sarcopenia
KFACS	Korean Frailty and Aging Cohort Study
AUC	Area under the curve
CI	Confidence interval
